# Extracellular vesicles orchestrate immune suppression and immune exclusion in cancer

**DOI:** 10.7150/ijbs.141013

**Published:** 2026-10-02

**Authors:** Seung-Jae Lee, Jaehoon Bae, Seong-Min Hong, Ji Young Hwang

**Affiliations:** 1 Functional Biomaterial Research Center, Korea Research Institute of Bioscience and Biotechnology, Jeongeup, 56212, Korea.; 2 Department of Applied Biotechnology, University of Science and Technology (UST), Daejeon, 34113, Korea.; 3 Functional Food Research Institute, Industry-university Cooperation Foundation, Daegu Haany University, Gyeongsan 38610, Korea.; 4 Department of Food Industry, Daegu Haany University, Gyeongsan, 38610, Korea.; 5 Division of Food Science and Technology and Institute of Agriculture and Life Science, Gyeongsang National University, Jinju, Gyeongnam 52828, Korea.; 6 Department of Food Science & Technology, College of Engineering, Dong-Eui University, Busan 47340, Korea.

Cancer immunotherapy has transformed modern oncology. Immune checkpoint blockade (ICB) targeting the PD-1/PD-L1 axis and adoptive cellular therapies have produced durable clinical responses across multiple malignancies, establishing immunotherapy as a cornerstone of cancer treatment 1. Nevertheless, a substantial proportion of patients either fail to respond or eventually develop acquired resistance. Increasing evidence indicates that therapeutic success is determined not only by the activation of tumor-reactive immune cells but also by the molecular and structural organization of the tumor microenvironment (TME) 1,2.

Traditionally, resistance to immunotherapy has been interpreted largely through the framework of immune suppression. Regulatory T cells, myeloid-derived suppressor cells (MDSCs), tumor-associated macrophages, inhibitory cytokines, metabolic stress, and immune-checkpoint signaling collectively impair antitumor immunity 1,2. Immune exclusion is an equally important determinant of treatment failure: even when tumor-reactive T cells are generated, they may remain confined to the tumor margin or surrounding stroma rather than entering malignant tissues 2. These processes are biologically intertwined because suppressive signaling networks can also promote stromal remodeling, extracellular matrix (ECM) deposition, and tissue stiffening. Restoring immune-cell activity alone may therefore be insufficient if physical barriers persist.

A key unresolved question is what mechanistically links immune suppression and immune exclusion within the TME. We propose that extracellular vesicles (EVs) represent an underappreciated link between these two forms of resistance. EVs are membrane-enclosed particles released by malignant, stromal, endothelial, and immune-cell populations. By transferring proteins, lipids, metabolites, messenger RNAs, microRNAs, and other regulatory molecules, EVs coordinate multidirectional communication across the TME 3,4. Their capacity to influence both immune and stromal compartments positions them to organize multiple barriers to antitumor immunity.

Compelling evidence supporting this framework was recently provided by Kim *et al*. 5. The authors developed ExoPERM, a pH-responsive PEGylated peptide platform that preferentially disrupts small EVs in the mildly acidic TME. ExoPERM restored CD8⁺ T-cell activity, attenuated cancer-associated fibroblast (CAF) activation, reduced stromal fibrosis, and enhanced cytotoxic T-cell infiltration 5. These coordinated effects extend the significance of ExoPERM beyond a single therapeutic platform and suggest that interrupting EV-mediated communication can alleviate functional immune suppression and physical immune exclusion simultaneously.

Within this functional-barrier framework, one of the best-characterized mechanisms of EV-mediated immune suppression involves EV-associated PD-L1. A foundational study demonstrated that PD-L1 can be released in vesicles of experimentally supported endosomal origin and can suppress T-cell activation beyond direct tumor-cell contact 6. These PD-L1-positive vesicles engage PD-1, inhibit T-cell receptor signaling, and impair proliferation and cytokine production. More recent work identified Munc13-4 as a regulator of PD-L1 sorting into and secretion through exosomes, directly linking vesicular cargo trafficking to tumor immune evasion 7. We use the broader terms “EV” or “small EV” throughout unless endosomal biogenesis was experimentally demonstrated; accordingly, “exosome” is retained only when describing studies that specifically established or mechanistically interrogated exosomal origin.

Kim *et al*. showed that ExoPERM-mediated EV disruption reduced PD-1/EV-associated PD-L1 interactions and restored CD8⁺ T-cell proliferation and effector activity 5. Tumor-associated EVs can also carry TGF-β, macrophage migration inhibitory factor (MIF), regulatory microRNAs, lipids, and other mediators that influence T-cell exhaustion, regulatory T-cell differentiation, MDSC accumulation, and M2-like macrophage polarization 3,4. These mechanisms are cargo-, recipient-cell-, and disease-context dependent rather than universal properties of all EVs.

The physical architecture of the TME is likewise shaped by reciprocal EV signaling. Cancer-derived vesicles can trigger fibroblast-to-myofibroblast differentiation through vesicular TGF-β and can promote α-smooth muscle actin expression, contractility, and collagen remodeling 8. Conversely, activated fibroblasts release EVs that can influence immune suppression, illustrating a feedback loop between stromal activation and immune regulation 3,4. CAF-derived EVs further regulate tumor-cell behavior, immune responses, and matrix organization 3,4. Thus, biochemical immunosuppression and structural immune exclusion can emerge as reciprocal outcomes of EV-mediated communication rather than as independent processes.

EV heterogeneity is central to interpreting this framework. The acidic TME contains overlapping EV populations from malignant cells, CAFs, endothelial cells, macrophages, dendritic cells, lymphocytes, and other stromal cells, with different biogenesis routes, surface markers, cargoes, and functions 3,4. Although many tumor-associated EV populations promote immune escape, others may transfer tumor antigens, support antigen presentation, or stimulate immune responses 3,4. It remains unclear whether ExoPERM preferentially affects malignant-cell-derived small EVs or broadly disrupts susceptible EV populations within acidic tumor regions. Broad disruption could be advantageous by simultaneously interrupting several protumor networks, but it could also remove beneficial antigen-presenting or immune-activating EV subsets. Defining the cellular origin and functional phenotype of ExoPERM-sensitive vesicles will therefore be important for patient selection and therapeutic optimization.

The fate of EV cargo after membrane disruption also warrants investigation. Rupture may expose proteins, lipids, metabolites, and nucleic acids to extracellular proteases, nucleases, lipases, phagocytic clearance, and renal or hepatic elimination. Rapid degradation would reinforce the therapeutic rationale, whereas persistence of bioactive cargo could produce unintended inflammatory, immunological, or coagulation-related effects. Translational studies should therefore quantify both intact vesicles and released cargo over time and determine whether membrane disruption neutralizes, redistributes, or transiently amplifies individual signals.

Spatial selectivity is another key consideration. ExoPERM exploits tumor acidity to limit systemic interference with physiological EV communication 5. However, acidic extracellular conditions also occur in inflamed, ischemic, infected, or injured tissues and in certain physiological niches. Off-target EV disruption in these environments cannot be excluded. Future development should integrate pharmacokinetic evaluation—including whole-body biodistribution, tumor penetration, and clearance of released cargo—with toxicological evaluation of repeated dosing, immunogenicity, and effects on normal EV-mediated communication. Studies in models that incorporate concomitant inflammation or tissue injury would help define the practical therapeutic window.

Clinical translation will additionally require reliable measurement of EV abundance, cellular origin, cargo composition, and biological activity. Current isolation procedures may co-isolate lipoproteins, protein aggregates, nucleoprotein complexes, and other extracellular nanoparticles, while bulk assays can obscure functionally distinct EV subsets. MISEV2023-compliant workflows, orthogonal characterization, and explicit reporting of preanalytical variables are therefore essential 9. Single-vesicle imaging, high-sensitivity or nano-flow cytometry, multiplexed surface-marker profiling, and single-particle molecular analysis can resolve heterogeneous populations that are averaged in bulk measurements 9,10. Applying these approaches before and after ExoPERM treatment could identify which vesicle populations are disrupted and which EV-associated biomarkers track pharmacodynamic response.

ExoPERM enhanced both anti-PD-1 therapy and adoptive T-cell transfer in preclinical models 5, supporting EV disruption as an enabling strategy rather than necessarily a standalone therapy. The same rationale extends selectively to additional modalities. Bispecific T-cell engagers and cancer vaccines can generate or recruit tumor-reactive T cells, but their activity may remain constrained by EV-mediated dysfunction and stromal exclusion. Radiotherapy and intratumoral immunostimulatory agents can increase antigen release and inflammatory signaling and may also alter EV abundance or cargo composition 3,4. Combining these approaches with spatially controlled EV disruption may preserve immune priming while reducing suppressive and exclusionary signals, but sequence, dose, and tumor context will need to be tested rather than assumed.

Taken together, tumor-associated EVs can act as communication hubs that couple immune regulation to tissue architecture. The relationship is not a simple linear cascade: tumor cells, EVs, CAFs, myeloid cells, lymphocytes, and the ECM participate in reciprocal feedback loops. Figure 1 therefore presents a restrained conceptual summary, while the reciprocal network and evidence boundaries are stated explicitly in the text and caption. This distinction avoids implying that every depicted cargo or recipient-cell pathway was established in the same experimental system.

In conclusion, the study by Kim *et al*. provides more than evidence for a novel EV-targeting strategy 5. It supports a framework in which EV-mediated communication links functional immune suppression with physical immune exclusion. The translational promise of this approach will depend on defining susceptible EV subpopulations, establishing the fate and safety of released cargo, validating selectivity in nonmalignant acidic tissues, and developing single-vesicle pharmacodynamic biomarkers. Under appropriate biological conditions, EV disruption may promote a more inflamed and T-cell-permissive TME and improve the efficacy of complementary immunotherapies.

## Figures and Tables

**Figure 1:**
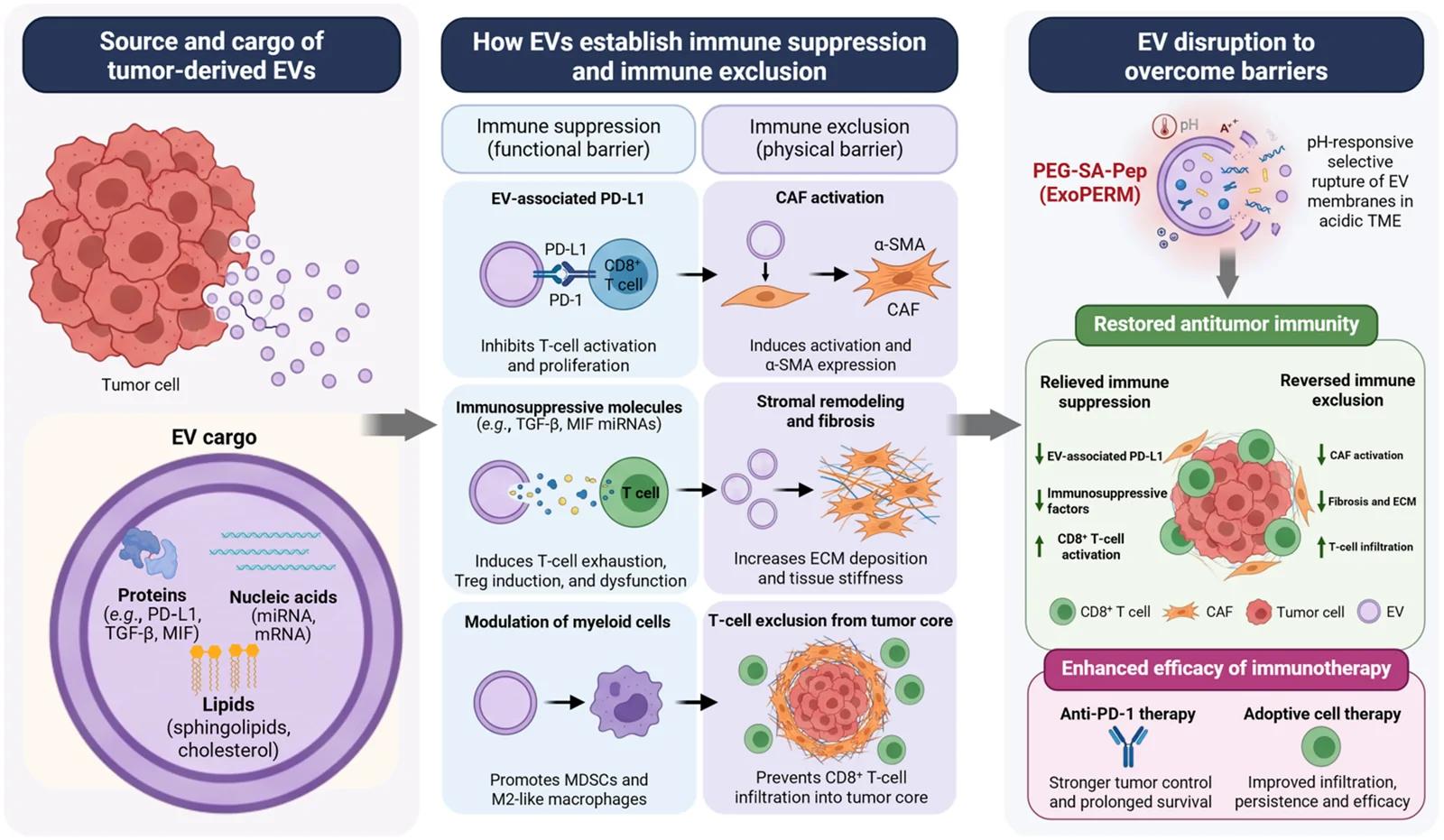
** Extracellular vesicles at the crossroads of immune suppression and immune exclusion in cancer.** Tumor-associated extracellular vesicles (EVs) carry proteins, lipids, metabolites, and regulatory nucleic acids that can suppress CD8⁺ T-cell function, promote immunosuppressive myeloid phenotypes, activate cancer-associated fibroblasts (CAFs), and enhance extracellular matrix (ECM) deposition and stromal restriction. These processes are interconnected through reciprocal signaling among tumor cells, EVs, CAFs, immune cells, and the ECM rather than forming a strictly linear sequence. In the highlighted preclinical study, the pH-responsive ExoPERM platform preferentially permeabilized small EVs within the acidic tumor microenvironment, attenuated EV-associated PD-L1 signaling and CAF activation, reduced fibrosis, and improved T-cell infiltration. Dashed return arrows from CAF activation and stromal remodeling to EV-mediated communication indicate proposed reciprocal feedback relationships, whereas solid arrows represent the principal pathways summarized in the figure. Not all depicted EV cargoes or recipient-cell mechanisms were directly evaluated in the ExoPERM study; the broader pathways are supported by the literature and may vary according to tumor context. Targeting tumor-associated EVs may therefore promote a more inflamed, T-cell-permissive tumor microenvironment and enhance responses to immune checkpoint blockade or adoptive cell therapy. However, the selectivity of this approach, the fate of released EV cargo, and its potential effects on beneficial EV subsets require further investigation. Created with BioRender.com.
